# CircRNA-100338 Is Associated With mTOR Signaling Pathway and Poor Prognosis in Hepatocellular Carcinoma

**DOI:** 10.3389/fonc.2019.00392

**Published:** 2019-05-14

**Authors:** Xiu-Yan Huang, Zi-Li Huang, Ping-Bao Zhang, Xin-Yu Huang, Jin Huang, Hong-Cheng Wang, Bin Xu, Jian Zhou, Zhao-You Tang

**Affiliations:** ^1^Department of General Surgery, Shanghai Jiaotong University Affiliated Sixth People's Hospital, Shanghai, China; ^2^Department of Radiology, Xuhui District Central Hospital of Zhongshan Hospital, Fudan University, Shanghai, China; ^3^Department of Urinary Surgery, Affiliated Hospital of Nantong University, Nantong, China; ^4^Department of Pathology, Shanghai Jiaotong University Affiliated Sixth People's Hospital, Shanghai, China; ^5^Department of General Surgery, The Tenth People's Hospital of Tongji University, Shanghai, China; ^6^Liver Cancer Institute and Zhongshan Hospital, Fudan University, Shanghai, China

**Keywords:** hepatocellular carcinoma, circRNA-100338, mTOR signaling pathway, REHB, EIF5

## Abstract

Hepatocellular carcinoma (HCC) is the leading cause of cancer-related deaths worldwide. Despite advances in the diagnosis and treatment of HCC, incidence, and mortality continue to rise. For accurate diagnosis and treatment of HCC, there is an urgent need to precisely understand the molecular mechanisms underlying HCC tumorigenesis and progression. Accumulating evidence showed that circRNAs, which are normally produced by scrambling of exons at the splicing process, are recognized as a novel class of endogenous noncoding RNA, which have microRNA sponging properties. In this study, we aim to investigate the circRNA-100338 mediated downstream pathway, and evaluate its association with clinicopathological parameters. Integrated analysis of circRNA-100338, miR-141-3p, and target genes revealed that RHEB, a key regulator in mTOR signaling pathway, was the target of miR-141-3p in hepatitis B-related HCC. CircRNA-100338 regulates the activity of mTOR signaling pathway *in vitro*. IHC analysis revealed that mTOR signaling pathway was more active in HCC tissues with elevated circRNA-100338 expression. These results indicated that circRNA-100338 could regulate mTOR signaling pathway through circRNA-100338/miR-141-3p/RHEB axis. Finally, correlation analysis of RHEB and EIF5 expression with clinicopathological parameters of HCC patients revealed that the circRNA-100338, RHEB, and EIF5 were indicators of poor prognosis in hepatitis B-related HCC. In conclusion, elevated circRNA-100338 activates mTOR signaling pathway in HCC via circRNA-100338/miR-141-3p/RHEB axis and associates with poor prognosis of hepatitis B-related HCC patients.

## Introduction

Hepatocellular carcinoma (HCC) is the leading cause of cancer-related deaths worldwide (1). Despite advances in the diagnosis and treatment of HCC, incidence and mortality continue to rise. For accurate diagnosis and treatment of HCC, there is an urgent need to precisely understand the molecular mechanisms underlying HCC tumorigenesis and progression. Currently, alpha fetoprotein (AFP) is widely used clinical biomarker for HCC diagnosis, while its sensitivity is only about 60% (2). Recently, sorafenib is approved as one of the few available targeted drugs recommended by definitive guides in clinical practice (3, 4) for advanced HCC, however, it is still limited in improving the overall survival of HCC patients (5, 6). In addition, other targeted drugs, such as sunitinib (7), brivanib (8), and everolimus (9), have been tested in clinical trials in the late years, but all failed in the third phase (10).

It is well-recognized that HCC is a heterogeneous disease of complicated etiology due to acquired gene mutations (11), epigenetic alterations (12), and dysregulation of coding or non-coding genes (13). For example, TERT promoter (54–60%), p53 (12–48%), β-catenin (11–37%), and Axin (5–15%) (14), have been identified as recurrently mutated genes in HCC. Moreover, DNA methylation profile of HCC revealed that MMP2, MMP9, and MMP12 were hypo-methylated in liver cancer using pyrosequencing (15). In addition, non-coding RNAs, such as long non-coding RNA (lncRNA) and microRNA (miRNA), have been widely recognized to contribute to HCC (16–19). Particularly, the long non-coding RNAs could provide signals of malignant transformation by interacting with chromatin, proteins and RNAs (20).

Accumulating evidences showed that circular RNAs (circRNAs), which are normally produced by scrambling of exons at the splicing process, are recognized as a novel class of endogenous noncoding RNA (21). For example, ciRS-7 has been shown to function as the sponge of miR-7, which could directly target several oncogenes and is involved in many different cancers (22). Other circRNAs also have microRNA (miRN sponging properties, even though most circRNAs are believed to possess other functions (23–25). In this study, we investigated the mechanism by which circRNA-100338 promoted tumor progression (26). As circRNA-100338 functions as an endogenous miRNA sponge for miR-141-3p (26), we investigated the circRNA-100338 mediated downstream pathway by an integrated data analysis, *in vitro* experiments, and protein expression in both HCC cell lines and tissues. The present study not only shed light on the downstream pathway mediated by circRNA-100338, but also provided a potential therapeutic target for HCC.

## Materials and Methods

### Clinical Specimens

A total 122 snap-frozen HCC tissues were obtained from the Hospital Clinic for immunohistochemistry analysis. All the experimental subjects were consecutive patients and did not receive any other treatment prior to operation. All HCC cases were confirmed by experienced pathologists. The survival rates at 1-, 3-, and 5-year were about 88.5% (108/122), 64.8% (79/122), and 54.9% (49/122). By the end of the follow-up, the overall survival rate was about 44.3% (54/122), and the pulmonary metastasis rate was about 40.2% (49/122). The clinicopathological parameters of the 122 HCC patients were summarized in Table 1.

**Table 1:** Correlation of RHEB and EIF5 expression with clinicopathological parameters of HCC patients.

**Features**		**RHEB and EIF5 expression**		***P***
		**Both high (*n* = 35)**	**Not both (*n* = 87)**	
Gender	Male	60%	73.56%	0.191
	Female	40%	26.44%	
Age, y	≤58	68.57%	66.67%	1.000
	>58	31.43%	33.33%	
Cirrhosis	Yes	62.86%	77.01%	0.121
	No	37.14%	22.99%	
AFP level, ng/mL	≤20	52.38%	31.03%	1.000
	>20	47.62%	68.97%	
γ-Glutamyl transferase, units/L	≤52	57.14%	63.22%	0.544
	>52	42.86%	36.78%	
Tumor size, cm	≤5	42.86%	43.68%	1.000
	>5	57.14%	56.32%	
Tumor number	Multiple	80%	52.87%	0.007
	Single	20%	47.13%	
Satellite	Yes	77.14%	44.83%	0.001
	No	22.86%	55.17%	
Encapsulation	Complete	14.29%	42.53%	0.003
	Incomplete	85.71%	57.47%	
Vascular invasion	Yes	82.86%	56.32%	0.007
	No	17.14%	43.68%	
Disease stage (TNM)	I-II	14.29%	34.48%	0.028
	III–IVA	85.71%	65.52%	
circRNA-100338 level	≤0.015	31.43%	72.41%	0.000
	>0.015	68.57%	27.59%	
Pulmonary metastasis^#^	Y	71.43%	27.59%	0.000
	N	28.57%	72.41%	

*HCC, hepatocellular carcinoma. ^#^Pulmonary metastasis was found during a 5-year follow-up. Significant difference: P < 0.05*.

### Gene, miRNA, and circRNA Expression Data

RNA-seq data of 40 matched samples (primary tumor and adjacent normal tissue) from 20 Chinese HCC patients were downloaded from Sequence Read Archive (SRA, www.ncbi.nlm.nih.gov/sra) database with accession number SRP069212 (27). The gene and miRNA expression data were downloaded from Gene Expression Omnibus (GEO, www.ncbi.nlm.nih.gov/geo/) database with accession numbers GSE77509 and GSE76903, respectively. For circular RNA identification and quantification, the RNA-seq reads were first mapped to the UCSC human reference genome (hg19) by BWA-meme aligner. The circular RNAs were predicted and quantified by CIRI-2 with GENCODE (28) annotation v19.

### MiRNA Target Prediction

The miRNA binding sites of circRNAs were predicted by Miranda (29) with option –*strict*. We selected default options for other parameters.

### Gene Set Overrepresentation Enrichment Analysis

The gene set overrepresentation enrichment analysis (ORA) was used to evaluate the enrichment of a gene list in a given gene set, such as pathway or a specific biological function. The ORA was implemented in WebGestalt (30) with hallmark gene set in MSigDB (31). The *P*-values for the hallmark gene sets were used to evaluate the significance of enrichment.

### HCC Cell Line and Cell Culture

At the authors' institution, a stepwise metastatic human HCC model system was established, which included a metastatic HCC model in nude mice LCI-D20, an HCC cell line MHCC97 with high metastatic potential that originated from LCI-D20 tumor (32), and cell clone MHCC97H from its parent MHCC97, with a lung metastatic rate up to 100% using orthotopic inoculation. The MHCC97H cells were maintained in antibiotic-free Dulbecco modified Eagle medium (DMEM, Gibco-BRL, Gaithersburg, MD). HCC cell lines of SMMC7721 with moderate invasiveness, BEL7402 with low invasiveness, and Hep3B with very low invasiveness were also prepared. The SMMC7721 and Hep3B cells were maintained in DMEM, while the BEL7402 cells were maintained in RPMI-1640 medium (Gibco, Gaithersburg, MD).

### MTT Cell Proliferation Assay

MTT (3-(4,5-dimethylthiazol-2-yl)-2,5-diphenyltetrazolium bromide) assay was used in this section (33) with modification. The untreated control or treated HCC cells (5 × 10^3^ cells/well) were used for MTT assay, which is based on the ability of a mitochondrial dehydrogenase enzyme from viable cells to cleave the tetrazolium rings of the pale yellow MTT and form a dark blue formazan crystals which is largely impermeable to cell membranes, thus resulting in its accumulation within healthy cells. Solubilization of the cells by the addition of a detergent results in the liberation of the crystals which are solubilized. The number of surviving cells is directly proportional to the level of the formazan product created. The color can then be quantified using a simple colorimetric assay. The results were read on a multiwell scanning spectrophotometer. The absorbance values were measured at a wavelength of 570 nm (with a reference of 630 nm).

### RNA Extraction and Quantitative Real-Time PCR

Total RNA from HCC cell lines was extracted using TRIzol and quantified using a NanoDrop ND-1000. The total RNA extracted from HCC cell lines, including both of MHCC97H and Hep3B cell lines with and without treatments, was reverse transcribed using random primers. The treatments included knockdown and overexpression of circRNA-100338. Quantitative PCR assays of cDNA were performed using a CFX96 Real-time PCR system (Bio-Rad) to quantify the target transcripts relative to the house-keeping genes U6 or GAPDH. Specifically, expression values in each sample were first assessed by qRT-PCR independently. A histogram was then generated based on the values from the independent measurements of all samples. Target cDNAs were amplified using the following probe set:

GAPDH_F: 5′-GGGAAACTGTGGCGTGAT-3′
GAPDH_R: 5′-GAGTGGGTGTCGCTGTTGA-3′
U6_F: 5′-GCTTCGGCAGCACATATACTAAAAT-3′
U6_R: 5′-CGCTTCACGAATTTGCGTGTCAT-3′
hsa_circRNA-100338_F:5′-AAAAGCAAGCAGTGCCCATA-3′
hsa_circRNA-100338_R:5′-GCTCGAATCAGGTCCACCA-3′
RHEB_F:5′-ACTCCTACGATCCAACCATAGA-3′
RHEB_R:5′-TGGAGTATGTCTGAGGAAAGATAGA-3′
EIF5_F:5′-GCG GCC GCA CCA TGG CAG ATG ACT TGG ACT TCG AG-3′
EIF5_R:5′-CGC AAG CTT CTA TTT TGC CAT GGC CTT GAT TGC-3′

### Knockdown and Overexpression of circRNA-100338

The small interfering RNA (siRNA) sequences were designed to target circRNA-100338. The siRNA was directed specifically against the backsplice. The detailed siRNA sequences were as follows:

(+) 5′-GUUUGUGGAACCACGUGAAUG-3′,
(–) 5′-UUCACGUGGUUCCACAAACUU-3′.

The circRNA-100338 overexpression was performed as our previous study described (26). To transcribe the circRNA-100338 transcript, a circRNA-100338 overexpression vector was constructed. The specially designed front and back circular frames were synthesized and added to pCDH-CMV-MCS-EF1-copGFP for circulation of the transcripts.

### Immunohistochemistry (IHC)

IHC was performed as previously described (34). We used anti-RHEB (Proteintech, Cat.No. 15924-1-AP) and anti-EIF5 antibodies (Proteintech, Cat.No. 11155-1-AP) to identify RHEB and EIF5, respectively. RHEB and EIF5 expression scores were calculated as the product of the cell staining percentage and cell staining intensity. The cell staining intensity was scored by four levels: (none) = 0, (weak) = 1, (medium) = 2, and (strong) = 3, and the cell staining percentage was scored as follows: <33% = 1, 33–66% = 2, and ≥66% = 3. The expression scores were calculated as the following equation:

Expression score = staining intensity × staining percentage Scores ≥3 were considered “high” scores, while lower scores were considered “low” scores. RHEB and EIF5 expression scores were determined independently by two researchers.

### Western Blot

Proteins were separated by 10% sodium dodecyl sulfate-polyacrylamide gel electrophoresis and transferred onto polyvinylidene difluoride membranes (Millipore, Bedford, MA). The membrane was blocked with 5% non-fat dried milk in TBST (20 mM Tris–HCl, 150 mM NaCl, and 0.1% Tween 20, pH 7.5) for 2 h and incubated overnight with antibodies against RHEB and EIF5 at 4°C. After washing with TBST buffer, membranes were incubated with horseradish peroxidase-conjugated antimouse IgG secondary antibodies for 1 h at room temperature and detected by enhanced chemiluminescence detection system (Amersham-Pharmacia Biotech, Braunschweig, Germany). GAPDH was used as an internal control (Santa-Cruz Biotechnologies, California, USA).

### Statistical Analysis

CircRNA expression normalization, subsequent data processing, and correlation analysis were performed using the R software package. The Spearman correlation between miRNA and gene/circRNA was calculated to evaluate the reverse expression pattern between miRNAs and their targets. The differential expression levels were evaluated by Wilcoxon rank-sum test. The survival analysis was performed using R *survival* package, and the samples were grouped by the optimal cutoff with the maximal AUC value in the Cox model.

## Results

### CircRNA-100338 Is Up-Regulated in HCC Tissues and Promotes Tumor Proliferation

**Figure 1:**
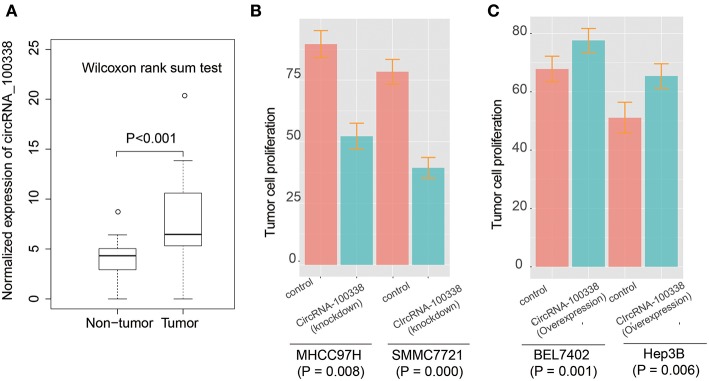
The expression patterns of circRNA-100338 and RHEB, and their expression correlation in HCC. **(A)** The expression levels of circRNA-100338 in non-tumor and tumor tissues. The differential expression levels are evaluated by Wilcoxon rank-sum test. **(B,C)** The tumor cell proliferation assays for HCC cell lines, including MHCC97H, SMMC7721, BEL7402, and Hep3B, with presence or absence of circRNA-100338.

To identify the expression patterns of circRNA-100338 in HCC and non-tumor tissues, we first collected RNA sequencing (RNA-seq) data of 40 samples (primary tumor and adjacent normal tissue) from 20 Chinese HCC patients from Sequence Read Archive (SRA) database with accession number SRP069212 (27). The circRNA expression profiles based on the RNA-seq data indicated that circRNA-100338 was significantly up-regulated in tumor tissues, as compared with non-tumor tissues (*P*-value < 0.001, Figure 1A). Cell proliferation assay demonstrated that knockdown of circRNA-100338 in highly metastatic potential HCC cell lines could significantly reduce the cancer cell proliferation (Figure 1B). Consistently, overexpression of circRNA-100338 in two HCC cell lines with low metastatic potential could increase cancer cell proliferation (*P* < 0.05, Figure 1C).

### Integrated Analysis of circRNA-100338, miR-141-3p, and Target Genes in Hepatitis B-Related HCC

**Figure 2:**
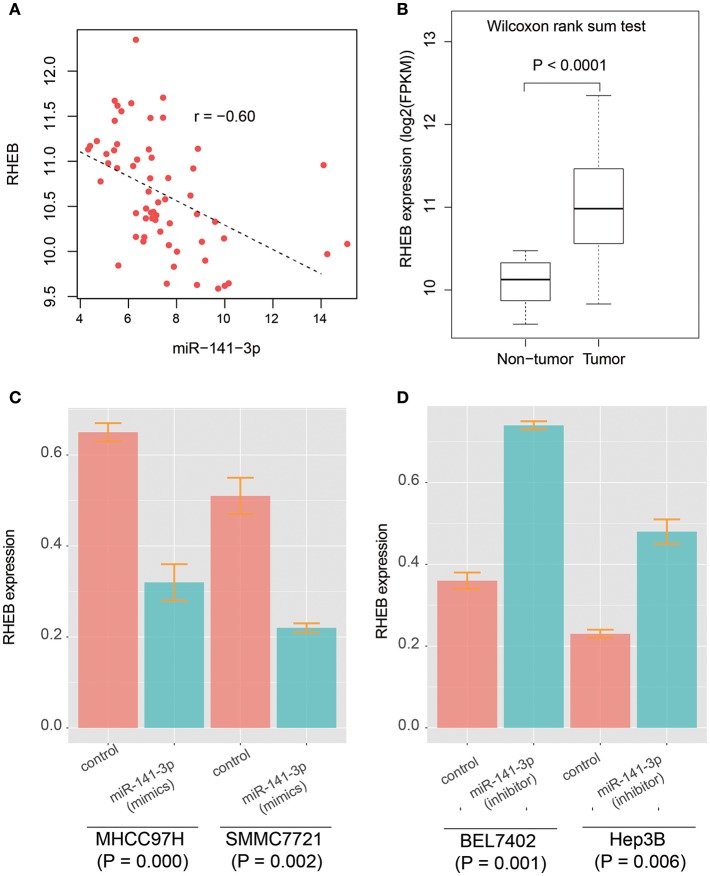
The prediction and validation of miR-141-3p binding with RHEB mRNA. **(A)** The RNA expression correlation between circRNA-100338 and RHEB. Each point in the scatterplot represents one sample. The expression of both RHEB and miR-141-3p are normalized and logarithm-transformed. Spearman correlation coefficient is used to identify the reverse expression correlation. **(B)** RHEB expression patterns in HCC tumor and non-tumor tissues. **(C,D)** RHEB expression in untreated and treated HCC cell lines by miRNA mimics or inhibitors.

It has been reported that circRNA-100338 has the potential to act as a competing endogenous RNA (ceRNA) by competing miR-141-3p with mRNAs by our previous study (26), however, the target genes regulated by circRNA-100338/miR-141-3p in HCC were still unknown. To identify the circRNA-100338 associated competing endogenous RNA network, we performed correlation analysis between miR-141-3p and 933 predicted target genes by miRanda using the RNA-seq data of the 40 samples (Supplementary Table 1). Finally, we only identified *RHEB* (Ras homolog enriched in brain) as the target of miR-141-3p in HCC (Figure 2A, Spearman correlation coefficient < −0.6), suggesting that circRNA-100338 may act as a ceRNA by competing with RHEB. Like circRNA-100338, RHEB was also up-regulated in tumor tissues (Figure 2B). These results suggested that miR-141-3p may negatively regulate RHEB, therefore, as circRNA-100338 may competitively bind with miR-141-3p, the upregulation of this circRNA would increase the RHEB RNA level. On the contrary, when circRNA-100338 was suppressed, miR-141-3p expression may be increased, which in turn increased the probability of its binding with RHEB, thus decreased RHEB expression.

### Validating the Binding of miR-141-3p With RHEB mRNA

As described in our previous study (26), the circRNA-100338 and RHEB were highly expressed, and miR-141-3p was lowly expressed in HCC cell lines with high metastatic potential, while the opposite expression patterns were observed in HCC cell lines with low metastatic potential. We then selected two HCC cell lines with high metastatic potential, MHCC97H, and SMMC7721, and two HCC cell lines with low metastatic potential, BEL7402, and Hep3B to investigate the regulatory relationship between circRNA-100338, miR-141-3p, and RHEB. We found that RHEB mRNA expression was significantly downregulated in MHCC97H (CI: 0.32 ± 0.04, *n* = 3) and SMMC7721 (CI: 0.22 ± 0.01, *n* = 3) cell lines with miR-141-3p mimics (*P* < 0.05, Figure 2C), as compared with controls (CI: 0.65 ± 0.02, *n* = 3 for MHCC97H, and CI: 0.51 ± 0.03, *n* = 3 for SMMC7721), respectively. Moreover, the RHEB mRNA expression was significantly upregulated in BEL7402 (CI: 0.74 ± 0.01, *n* = 3) and Hep3B (CI: 0.48 ± 0.03, *n* = 3) cell lines with miR-141-3p inhibitor (*P* < 0.05, Figure 2D), as compared with the controls (CI: 0.36 ± 0.02, *n* = 3 for BEL7402, and CI: 0.23 ± 0.01, *n* = 3 for Hep3B), respectively. These results demonstrated that RHEB was a target of miR-141-3p.

### CircRNA-100338 Regulates the Activity of mTOR Signaling Pathway *in vitro*

It is well-recognized that RHEB acts as an activator of mTOR signaling pathway (35, 36). The integrated analysis provided us a hint that circRNA-100338 may regulate mTOR signaling pathway by increasing *RHEB* RNA expression. Gene set overrepresentation enrichment analysis (ORA) revealed that the highly correlated genes with circRNA-100338 (Spearman correlation coefficient > 0.5) was significantly enriched in mTORC1 signaling (FDR = 9.36e-5, Table 2), further demonstrating that circRNA-100338 may participate in mTOR signaling pathway.

**Table 2:** The pathways enriched by genes highly correlated with circRNA-100338.

**Description**	**#Genes**	***P*-value**	**FDR**
Cell cycle progression: E2F targets	26	1.78E-15	8.88E-14
Cell cycle progression: G2/M checkpoint	17	1.99E-07	4.98E-06
mTORC1 signaling	15	5.62E-06	9.36E-05
MYC targets, variant 1	14	2.64E-05	3.30E-04
Unfolded protein response; ER stress	9	3.17E-04	3.17E-03

*Spearman correlation coefficient > 0.5, and ORA test, FDR < 0.05*.

**Figure 3:**
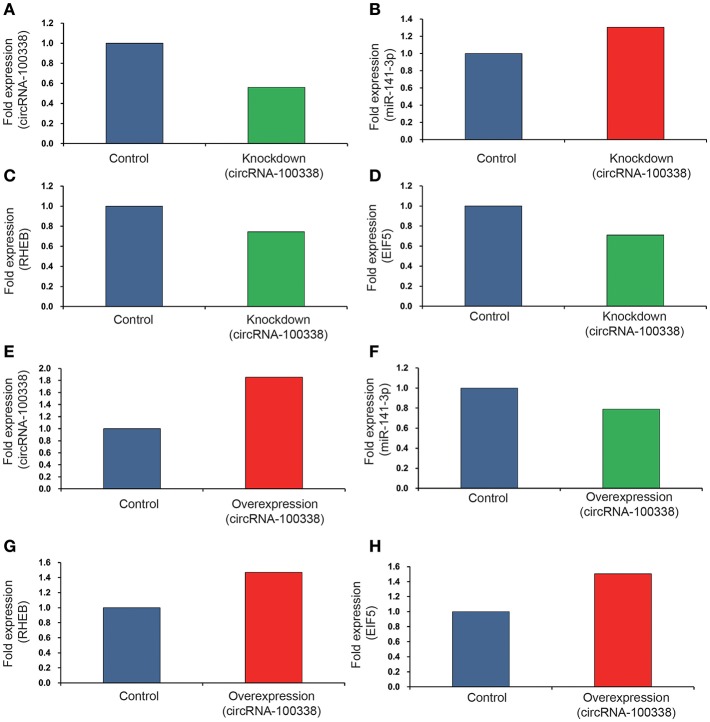
The RNA expression levels of circRNA-100338, miR-141-3p, RHEB, and EIF5 in HCC cell lines with untreated, knockdown, and overexpression of circRNA-100338. The RNA expression levels of circRNA-100338, miR-141-3p, RHEB, and EIF5 in MHCC97H cells with untreated and knockdown of circRNA-100338 are displayed in **(A–D)**, while those in Hep3B cells with untreated and overexpression of circRNA-100338 are displayed in **(E–H)**. The blue, green and red bars represent the controls, down-regulations and up-regulations, respectively.

Since circRNA-100338 was upregulated in HCC, we used small interference RNAs (siRNAs) to knock down the expression of circRNA-100338 in an HBV-related HCC cell line MHCC97H, in which circRNA-100338 was highly expressed. qRT-PCR analysis revealed that circRNA-100338 was decreased by 44%, demonstrating that the inhibition was successful (Figure 3A). In contrast, miR-141-3p was up-regulated about 30.3% in the knockdown (KD) MHCC97H cell line (KD group) compared with the control (Figure 3B). To examine the activity of mTOR signaling pathway, we also measured the RNA expression of RHEB and EIF5 (eukaryotic translation initiation factor-5), two key regulators in mTOR signaling pathway (37). In accordance with the downregulation of circRNA-100338, down-regulation of RNA expression for RHEB (25.4%) and EIF5 (29.1%) was also observed in KD group (Figures 3C,D). These results suggested that inhibition of circRNA-100338 could weaken the activity of mTOR signaling pathway.

To further explore the role of circRNA-100338 in mTOR signaling pathway, we also overexpressed (OE) circRNA-100338 in Hep3B cell line (OE group), in which circRNA-100338 was lowly expressed. As expected, we found that circRNA-100338 was significantly up-regulated in the OE cell lines (85.7%, Figure 3E). The miR-141-3p was reduced about 21.1% (Figure 3F). RHEB and EIF5 were also observed up-regulated in OE group as compared with the control (Figures 3G,H), indicating that up-regulation of circRNA-100338 could promote the activation of mTOR signaling pathway.

**Figure 4:**
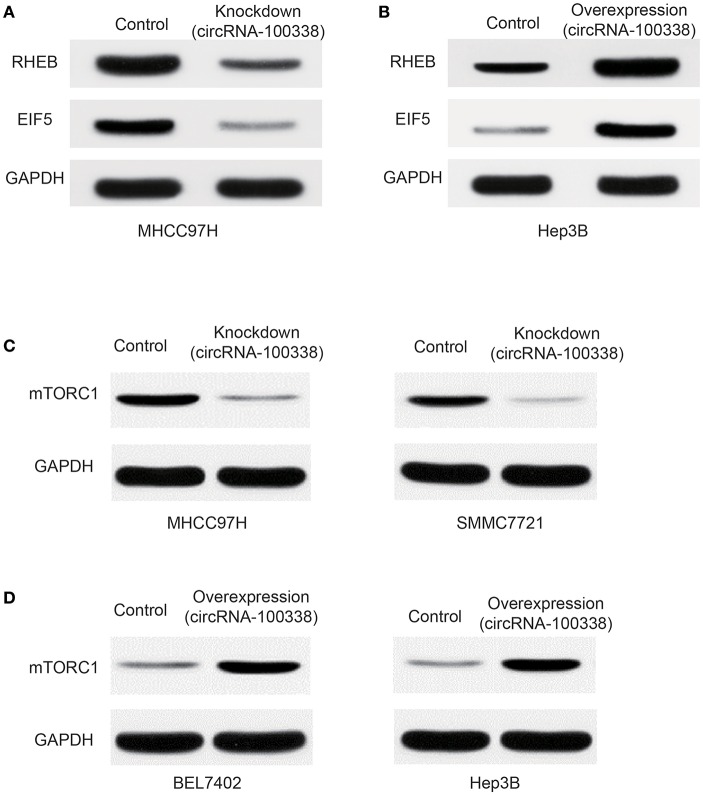
Protein expression of RHEB, EIF5, and mTOR complex-1 (mTORC1) in untreated, and circRNA-100338-overexpressed or –knocked down HCC cell lines. The protein expression of RHEB and EIF5 in cell lines with knockdown **(A)** and overexpression **(B)** of circRNA-100338 was quantified by western blot analysis. **(C)** mTORC1 protein expression in cell lines untreated and knocked down by circRNA-100338. **(D)** mTORC1 protein expression in cell lines untreated and overexpressed by circRNA-100338.

Furthermore, western blot analysis demonstrated that RHEB and EIF5 were down-regulated in cell line with knockdown of circRNA-100338 (Figure 4A), and up-regulated in cell line with circRNA-10338 overexpression when compared with the control (Figure 4B). To further demonstrate the role of circRNA-100338 in mTOR signaling, we also measured the mTORC1 (mTOR complex 1) protein expression in the cell lines above and another pair of highly and lowly metastatic potential HCC cell lines, MHCC97H, and BEL7402, in the presence and absence of circRNA-100338, and found that mTORC1 could be upregulated by the circRNA-100338 (Figures 4C,D). These results disclosed that circRNA-100338 could regulate the activity of mTOR signaling pathway through miR-141-3 p.

### mTOR Signaling Pathway Was More Active in HCC Tissues With Elevated circRNA-100338 Expression

**Figure 5:**
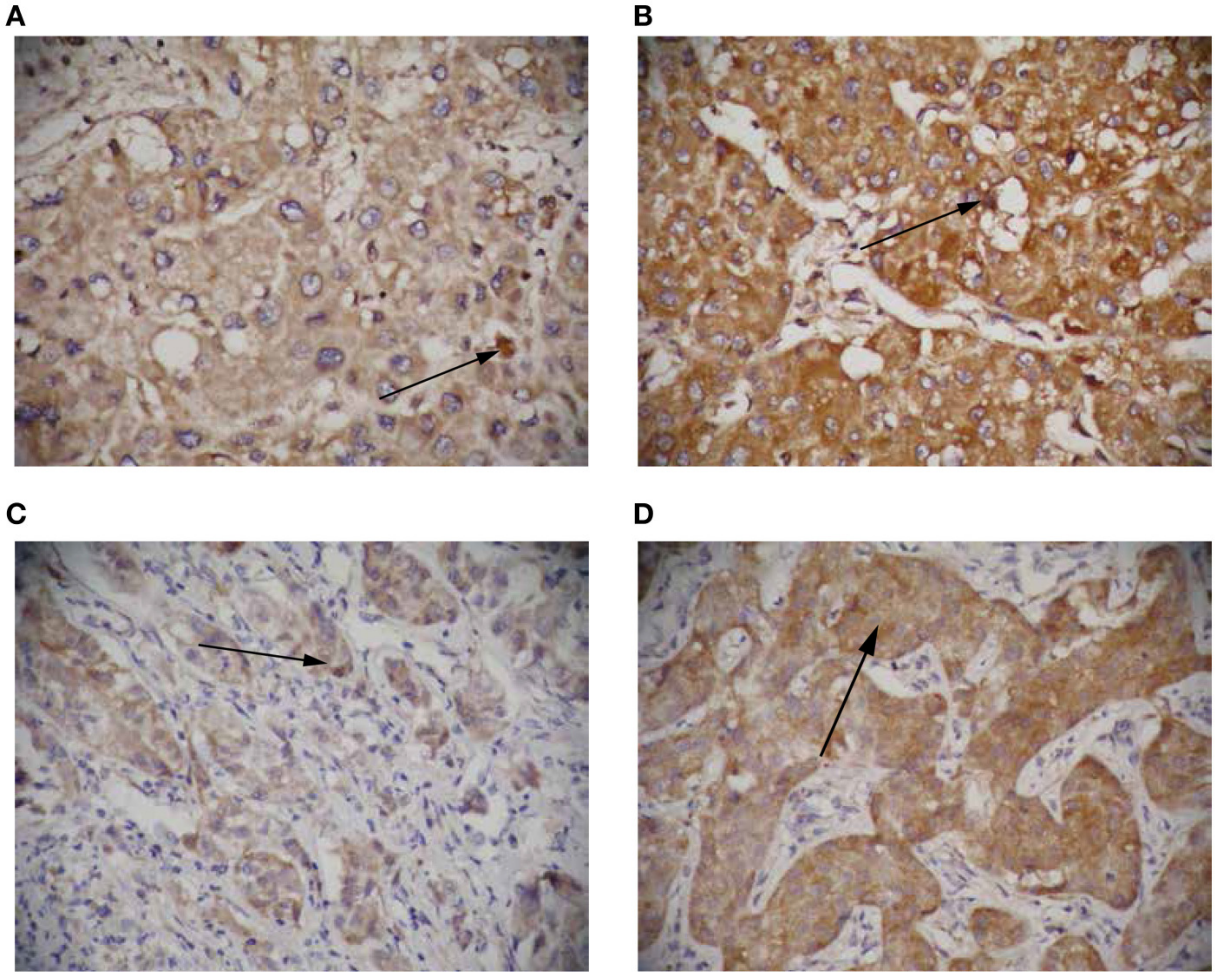
The RHEB and EIF5 protein expression in HCC tissues with high and low circRNA-100338 expression. Representative images showing RHEB **(A,B)** and EIF5 **(C,D)** IHC staining patterns in HCC with high **(B,D)** and low **(A,C)** expression of circRNA-100338 (original magnification, × 400).

To further elucidate the role of circRNA-100338 in the regulation of mTOR signaling pathway, we collected 122 tumor samples of hepatitis B-related HCC patients. The patients were then split by circRNA-100338 expression in HCC: >0.015 (circRNA-100338 high expression group, *n* = 48); and ≤0.015 (circRNA-100338 low expression group, *n* = 74). Qualitative analysis of RHEB and EIF5 protein expression by immunohistochemistry (IHC) revealed that observed expression levels of these two proteins were higher in circRNA-100338 high expression group than in circRNA-100338 low expression group (Figures 5A–D). In accordance with HCC cell lines with overexpression of circRNA-100338, hepatitis B-related HCC tissues in circRNA-100338-high group also showed high activity of mTOR signaling pathway, further demonstrating that circRNA-100338 could promote activation of mTOR signaling pathway.

### Clinical Impact of mTOR Signaling Pathway in Hepatitis B-Related HCC

To investigate the clinical impact of the two regulators, RHEB, and EIF5, of mTOR signaling in hepatitis B-related HCC, we evaluated the association of the two proteins with the clinicopathological parameters, which was summarized in Table 1. We observed that the pulmonary metastasis rate was significantly higher in samples with both overexpression of RHEB and EIF5 than in the remaining 87 samples (*P* = 0.000, 71.4 vs. 27.6%). Similarly, vascular invasion rate was also observed to be higher in samples with both overexpression of RHEB and EIF5 than in the remaining 87 samples (*P* = 0.007, 80.6 vs. 56.3%, Table 1), indicating that the activation of mTOR signaling pathway by circRNA-100338 could result in high probability of pulmonary metastasis and/or vascular invasion, further suggesting that the mTOR signaling pathway activated by circRNA-100338/miR-141-3p/RHEB may promote HCC pulmonary metastasis.

**Figure 6:**
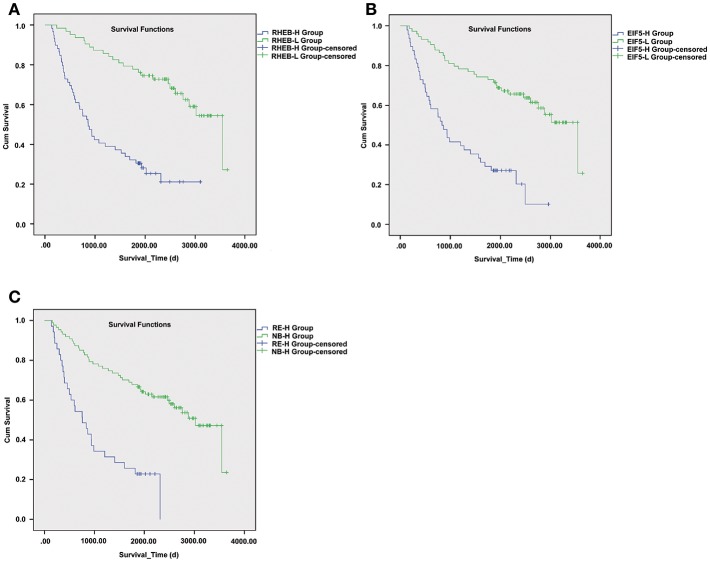
The survival differences between samples with one or both of RHEB and EIF5 elevated expression. The Kaplan–Meier (KM) curves for samples with high (RHEB-H or EIF5-H group) and low (RHEB-L or EIF5-L group) expression of RHEB or EIF5 are illustrated in **(A)** and **(B)**, respectively. **(C)** The KM curves represent the samples in high expression of both RHEB and EIF5 (RE-H group) and those in low expression of one of the two proteins (RE-L group). The survival analysis indicates the prognostic values of RHEB and EIF5 in HCC.

As upregulation of circRNA-100338 was associated with short overall survival based on our previous study, we also evaluated the association between HCC overall survival and expression levels of one or both of the RHEB and EIF5. As expected, short overall survival was also observed in HCC samples with one or both of elevated expression of RHEB and EIF5 (Figure 6), indicating that the activation of mTOR signaling pathway was positively correlated with short overall survival.

## Discussion

HCC is a heterogeneous disease of complicated etiology. The molecular basis of HCC about protein-coding genes has largely been studied in the context of tumorigenesis, progression, and metastasis. However, circRNAs, a novel class non-coding RNAs are characterized to act as cancer driver RNAs, and understanding their dysregulation and regulatory mechanisms can promote the development of new diagnostic or therapeutic strategies.

As our previous study reported, circRNA-100338 is closely correlated with a low cumulative survival rate and metastatic progression in HCC patients with hepatitis B. In the present study, we investigated the mechanism by which circRNA-100338 promoted tumor progression. As circRNA-100338 functions as an endogenous sponge for miR-141-3p, an integrated analysis of circRNA-100338, miR-141-3p, and target genes was conducted (26). We identified RHEB as the target of miR-141-3p in HCC, suggesting that circRNA-100338 may act as a ceRNA by competing with RHEB, which could activate the protein kinase activity of mTORC1, thereby play a key role in the regulation of mTOR signaling. We thus speculated that elevated expression of circRNA-100338 might activate mTOR signaling pathway. Further experiments *in vitro* by circRNA-100338 knockdown in MHCC97H and overexpression in Hep3B demonstrated that circRNA-100338 could regulate RHEB through miR-141-3p. Moreover, we also observed that mTORC1 and EIF5, which were two key regulators in mTOR signaling pathway, and controlled the protein synthesis initiation, was also regulated by the circRNA-100338/miR-141-3p/RHEB axis. In accordance with experiments *in vitro*, hepatitis B-related HCC tissues in circRNA-100338-high group also showed elevated expression of both RHEB, mTORC1, and EIF5, further demonstrating that circRNA-100338 could promote activation of mTOR signaling pathway. More importantly, activated mTOR signaling pathway by circRNA-100338/miR-141-3p/RHEB axis showed high correlation with poor prognosis in hepatitis B-related HCC. Particularly, the pulmonary metastasis rate was higher, and the overall survival was shorter in hepatitis B-related HCC with elevated expression of one or both of RHEB and EIF5 (*P* < 0.001).

As annotated by circBase (38), circRNA-100338 was originated from *SNX27* gene, which produced a total of 22 circRNA isoforms in human tissues or cells. In addition to circRNA-100338, hsa_circ_0014186, another circRNA isoform of *SNX27*, was down-regulated in human plasma with active tuberculosis (39), however, the function of other circRNA isoforms was still unknown, suggesting that further studies were needed to characterize the FUNCTION of circRNAs.

The eukaryotic translation initiation factors (eIFs), the downstream effector of mTOR signaling pathway, are involved in the initiation step of protein translation (40). Each eIF has its unique role in the initiation step. In this study, we observed one of the eIFs, EIF5, was regulated by circRNA-100338/miR-141-3p/RHEB axis, which was consistent with previous study that high expression of EIF5 had a significant influence on survival in patients with HBV (37).

There are some limitations in this study. The lack of *in vivo* data to support the role of circRNA-100338 in mTOR signaling activation is the major limitation. Moreover, we cannot exclude the possibility that other downstream pathways of RHEB might also mediate HCC metastasis. Despite these limitations, we believe that the current study provides preliminary and powerful data underscoring the value of circRNA-100338/miR-141-3p/RHEB axis in HCC diagnosis and treatment.

Overall, circRNA-100338 is closely associated with poor prognosis of hepatitis B-related HCC by activating mTOR signaling pathway via circRNA-100338/miR-141-3p/RHEB axis. This study makes the connection between circRNA-100338 and mTOR signaling pathway in HCC cells and may provide a potential therapeutic target for HCC.

## Ethics Statement

This study was approved by the Research Ethics Committee of Shanghai Jiaotong University affiliated Sixth People's Hospital, and informed consent was obtained from each patient. All specimens were collected in the operating room immediately (≤15 min) after tissue removal and were snap frozen in liquid nitrogen and stored at −80°C. All methods were performed in accordance with the relevant guidelines and regulations. Participants gave their written informed consent for the materials to appear in publications without limit on the duration of publication.

## Author Contributions

X-YaH, Z-LH, P-BZ, X-YuH, JH, and Z-YT conceived and designed the experiments. X-YaH, Z-LH, P-BZ, X-YuH, and JH performed the experiments. X-YaH, Z-LH, X-YuH, H-CW, BX, and JZ analyzed the data. X-YaH, Z-LH, H-CW, BX, JZ, and Z-YT contributed reagents, materials, analysis tools. X-YaH, Z-LH, P-BZ, X-YuH, JH, and Z-YT wrote the paper. All authors read and approved the final manuscript.

### Conflict of Interest Statement

The authors declare that the research was conducted in the absence of any commercial or financial relationships that could be construed as a potential conflict of interest.
